# Supplementary material for: Whole Exome Sequencing Reveals Novel Candidate Genes in Familial Forms of Glaucomatous Neurodegeneration
Source: Genes (Basel). 2023 Feb 15;14(2):495. doi: 10.3390/genes14020495 (PMC9957298; doi:10.3390/genes14020495)
Supplement: Supplementary file 1 [file genes-14-00495-s001.zip › Narta et al Supplementary_Figures Binder.pdf]

Figure S1 *MYOC mutation screening*

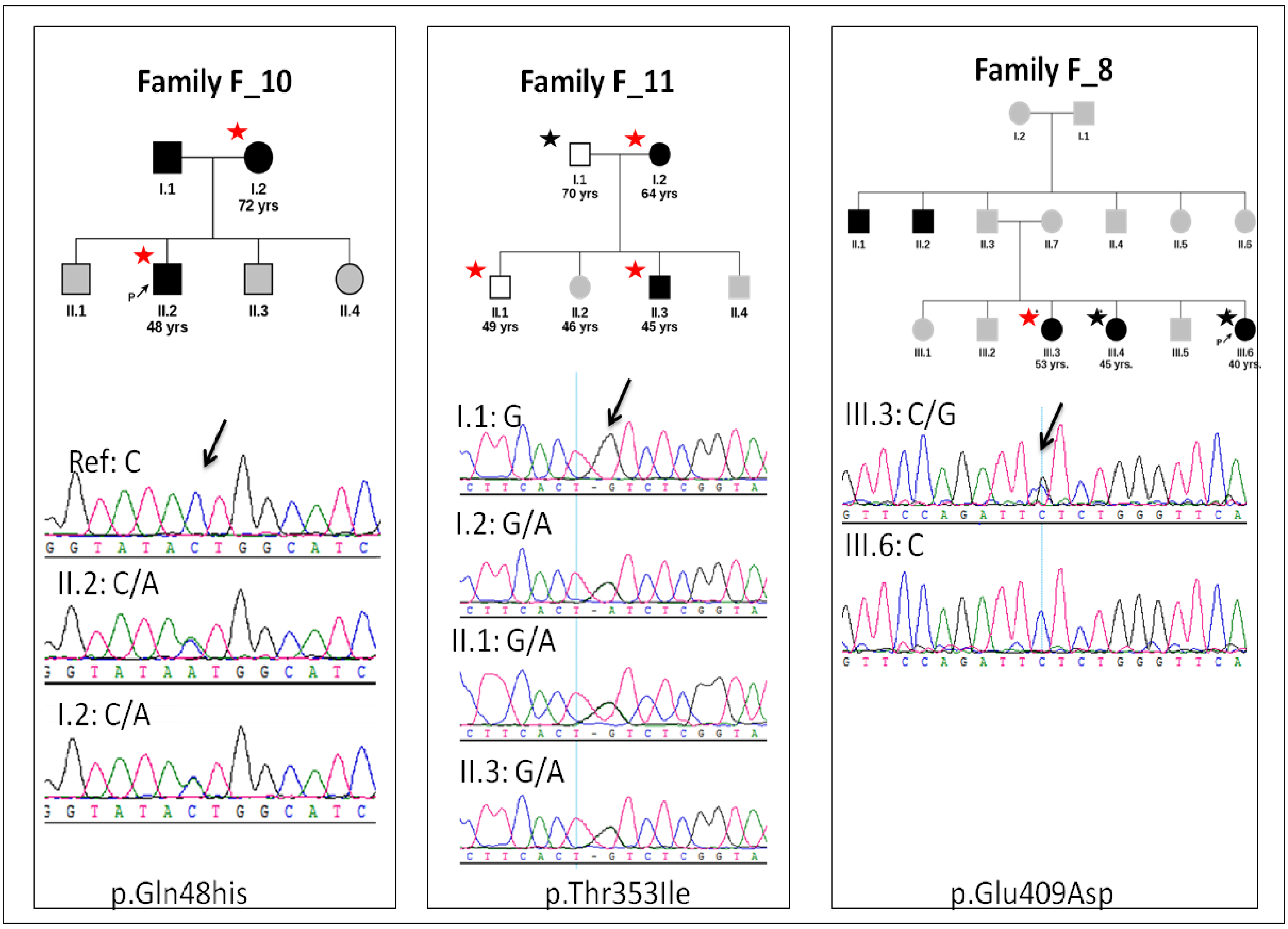

Figure S2a

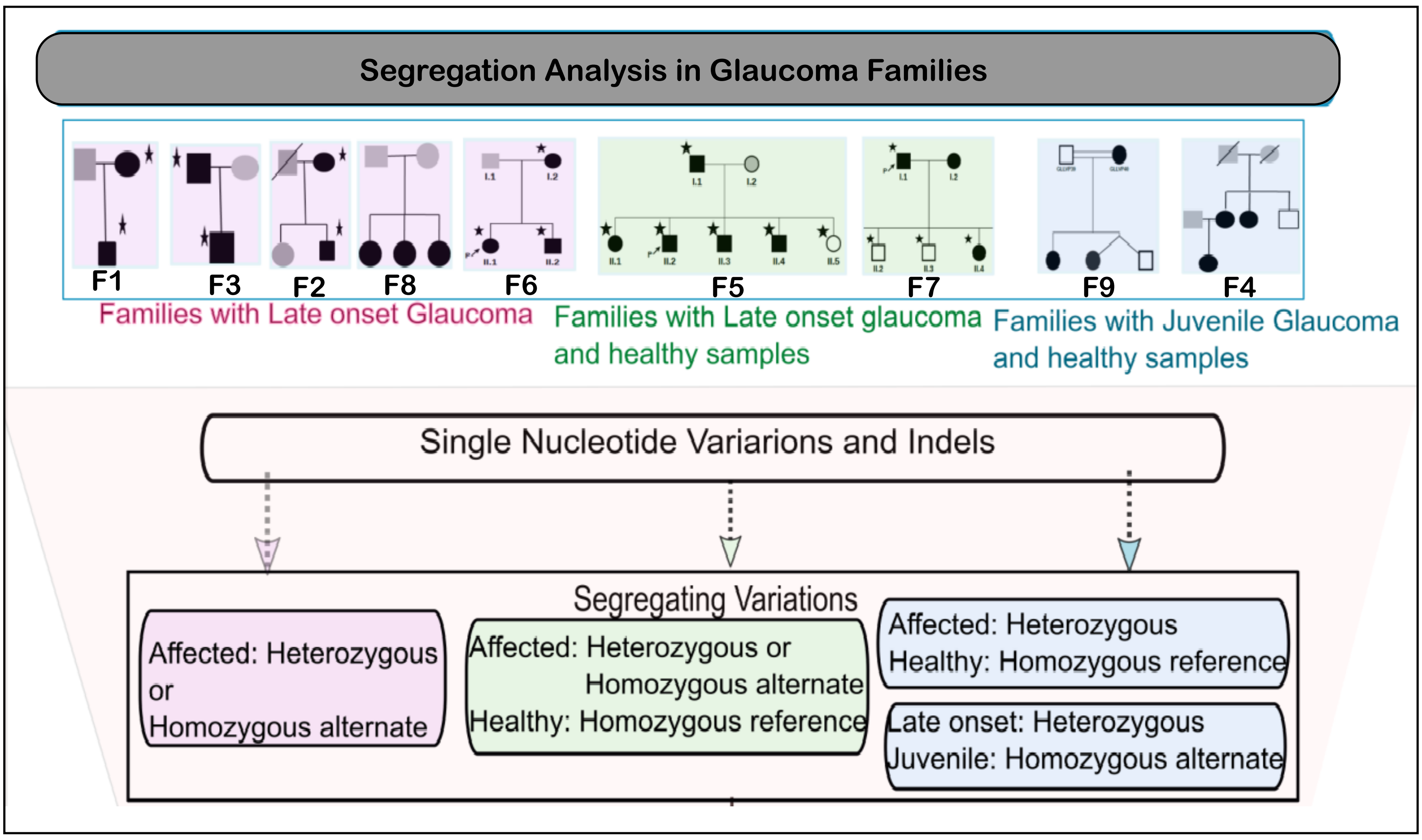

Segregation Analysis in Glaucoma Families
F1
F3
F2
F8
F6
F5
F7
F9
F4
Families with Late onset Glaucoma
Families with Late onset glaucoma and healthy samples
Families with Juvenile Glaucoma and healthy samples
Single Nucleotide Variarions and Indels
Segregating Variations
Affected: Heterozygous or Homozygous alternate
Affected: Heterozygous or Homozygous alternate
Healthy: Homozygous reference
Affected: Heterozygous
Healthy: Homozygous reference
Late onset: Heterozygous
Juvenile: Homozygous alternate

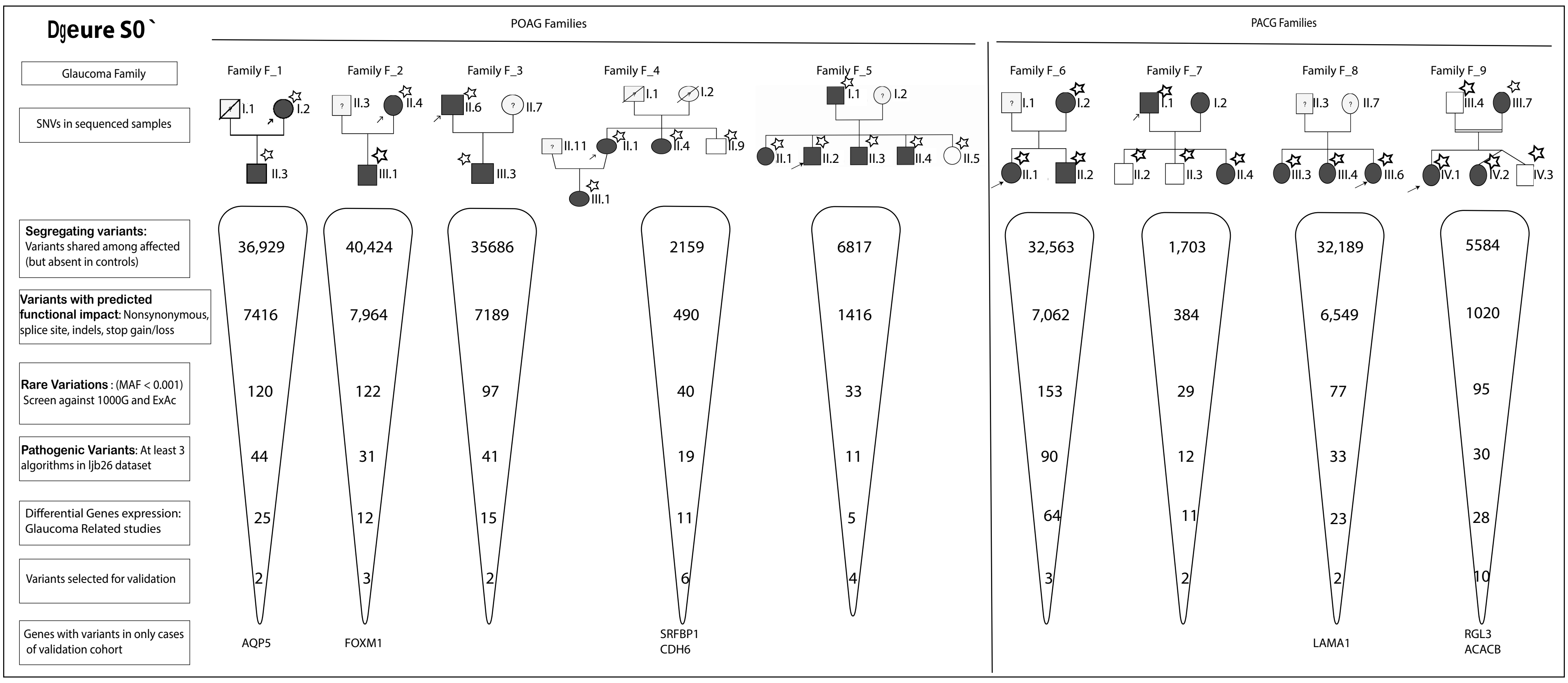
Dgeure S0`
POAG Families
PACG Families
Glaucoma Family
SNVs in sequenced samples
Segregating variants: Variants shared among affected (but absent in controls)
Variants with predicted functional impact: Nonsynonymous, splice site, indels, stop gain/loss
Rare Variations : (MAF < 0.001) Screen against 1000G and ExAc
Pathogenic Variants: At least 3 algorithms in ljb26 dataset
Differential Genes expression: Glaucoma Related studies
Variants selected for validation
Genes with variants in only cases of validation cohort
Family F_1
I.1
I.2
II.3
36,929
7416
120
44
25
2
AQP5
Family F_2
II.3
II.4
III.1
40,424
7,964
122
31
12
3
FOXM1
Family F_3
II.6
II.7
III.3
35686
7189
97
41
15
2
Family F_4
I.1
I.2
II.11
II.1
II.4
II.9
III.1
2159
490
40
19
11
6
SRFBP1
CDH6
Family F_5
I.1
I.2
II.1
II.2
II.3
II.4
II.5
6817
1416
33
11
5
4
Family F_6
I.1
I.2
II.1
II.2
32,563
7,062
153
90
64
3
Family F_7
I.1
I.2
II.2
II.3
II.4
1,703
384
29
12
11
2
Family F_8
II.3
II.7
III.3
III.4
III.6
32,189
6,549
77
33
23
2
LAMA1
Family F_9
III.4
III.7
IV.1
IV.2
IV.3
5584
1020
95
30
28
10
RGL3
ACACB

Figure S3a

POAG Families

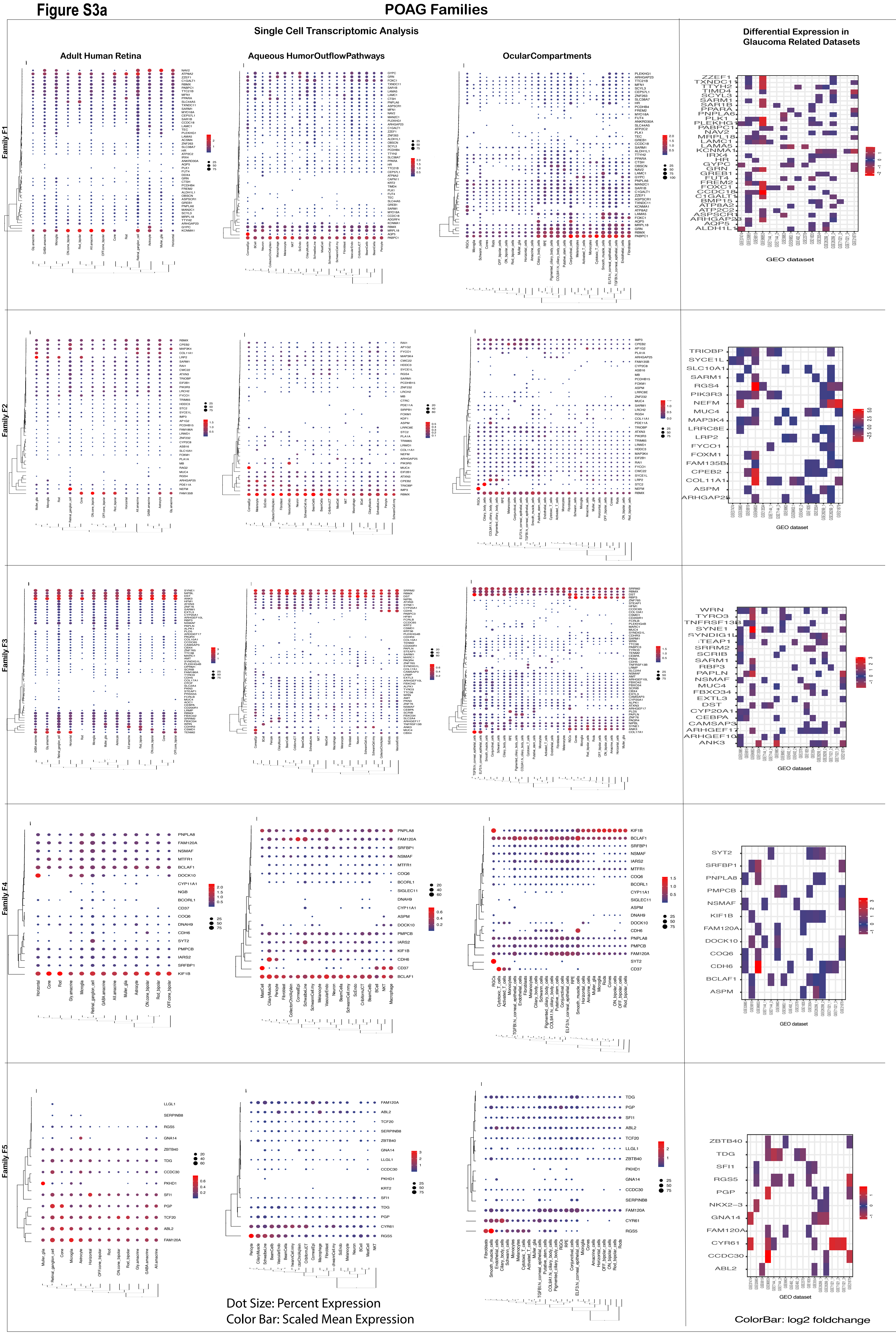

Figure S3b

PACG Families

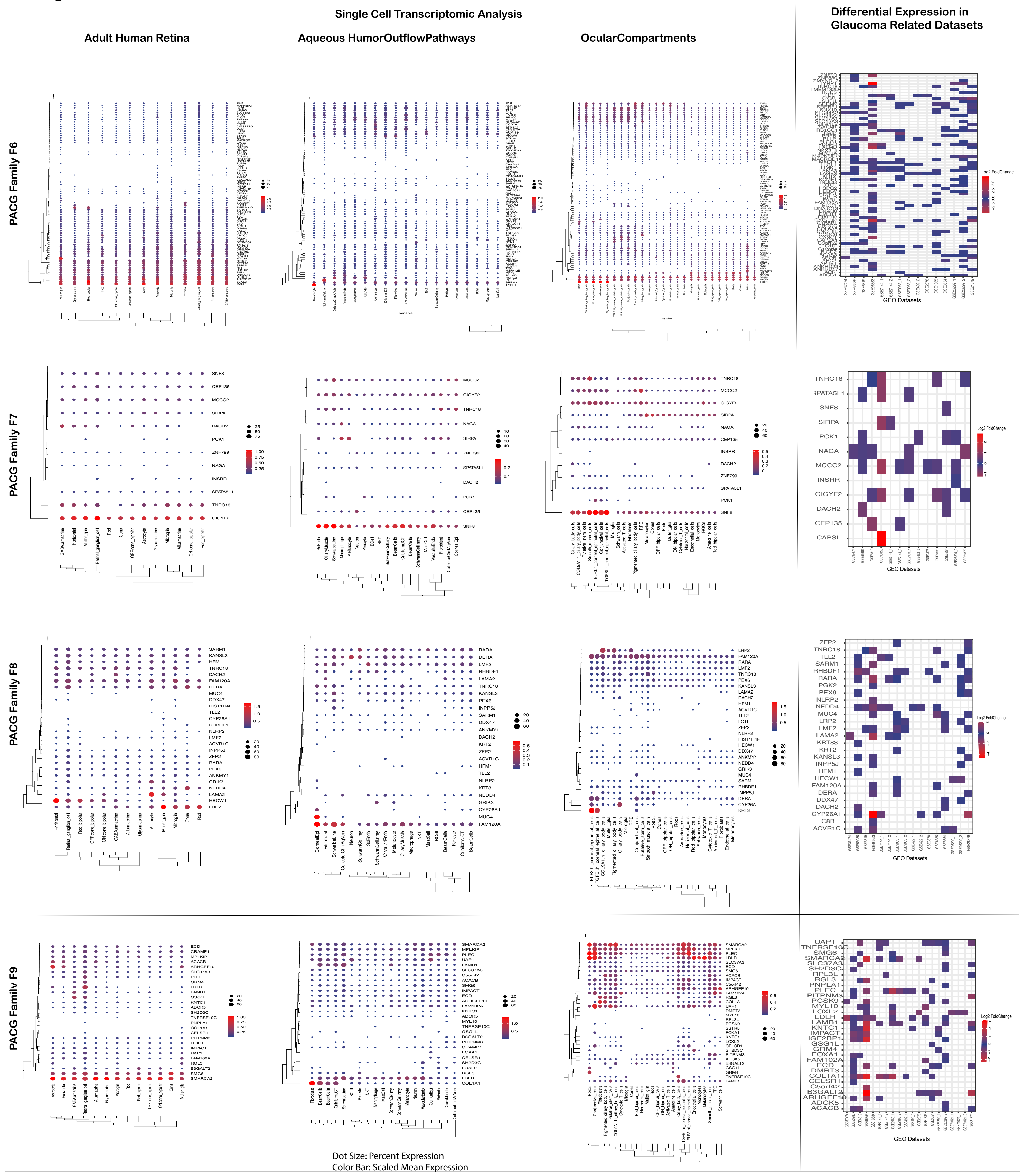

Figure S4

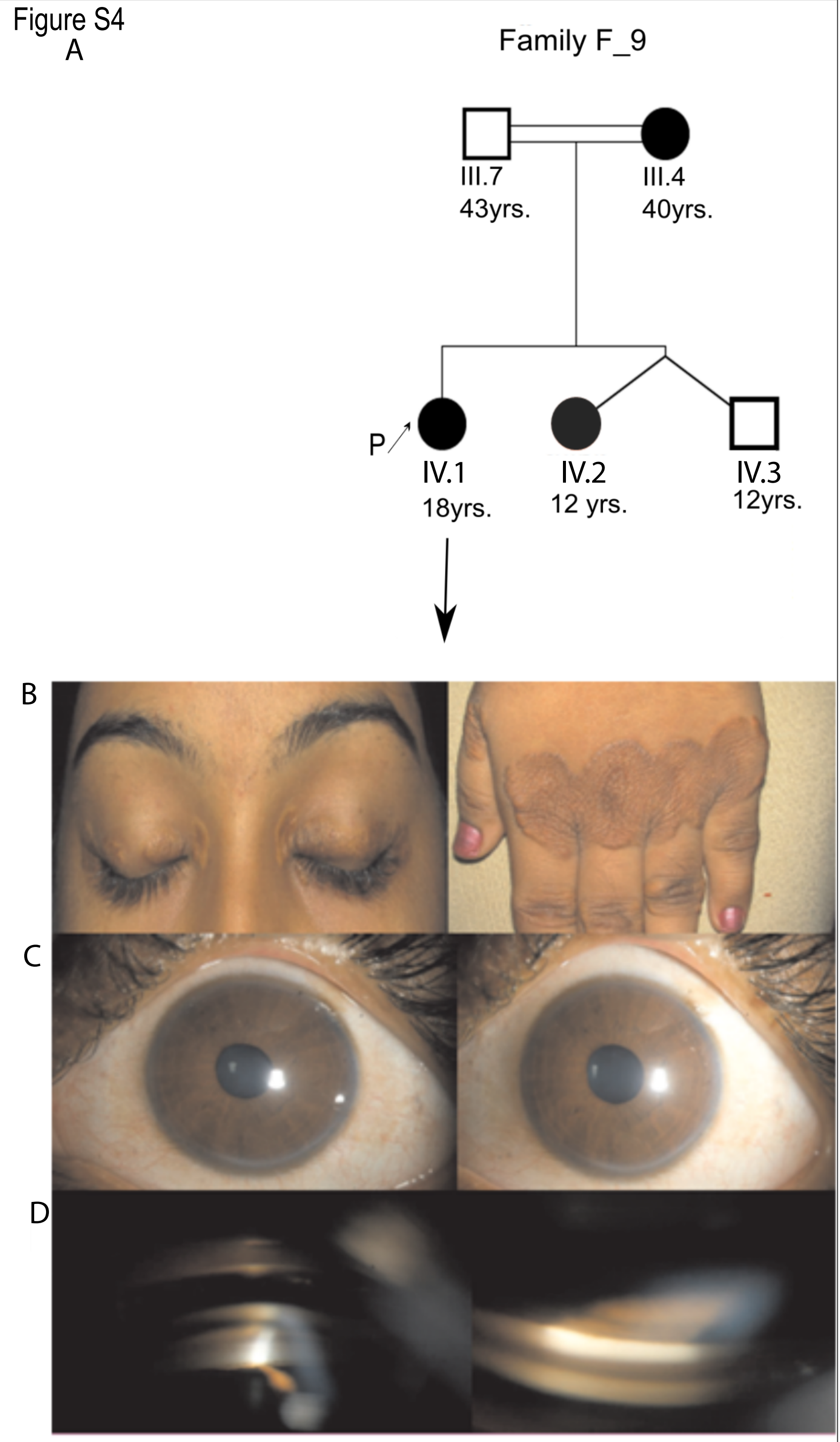
A
Family F_9
III.7
43yrs.
III.4
40yrs.
P
IV.1
18yrs.
IV.2
12 yrs.
IV.3
12yrs.
B
C
D

Figure S5. **Sanger Sequencing Validations**

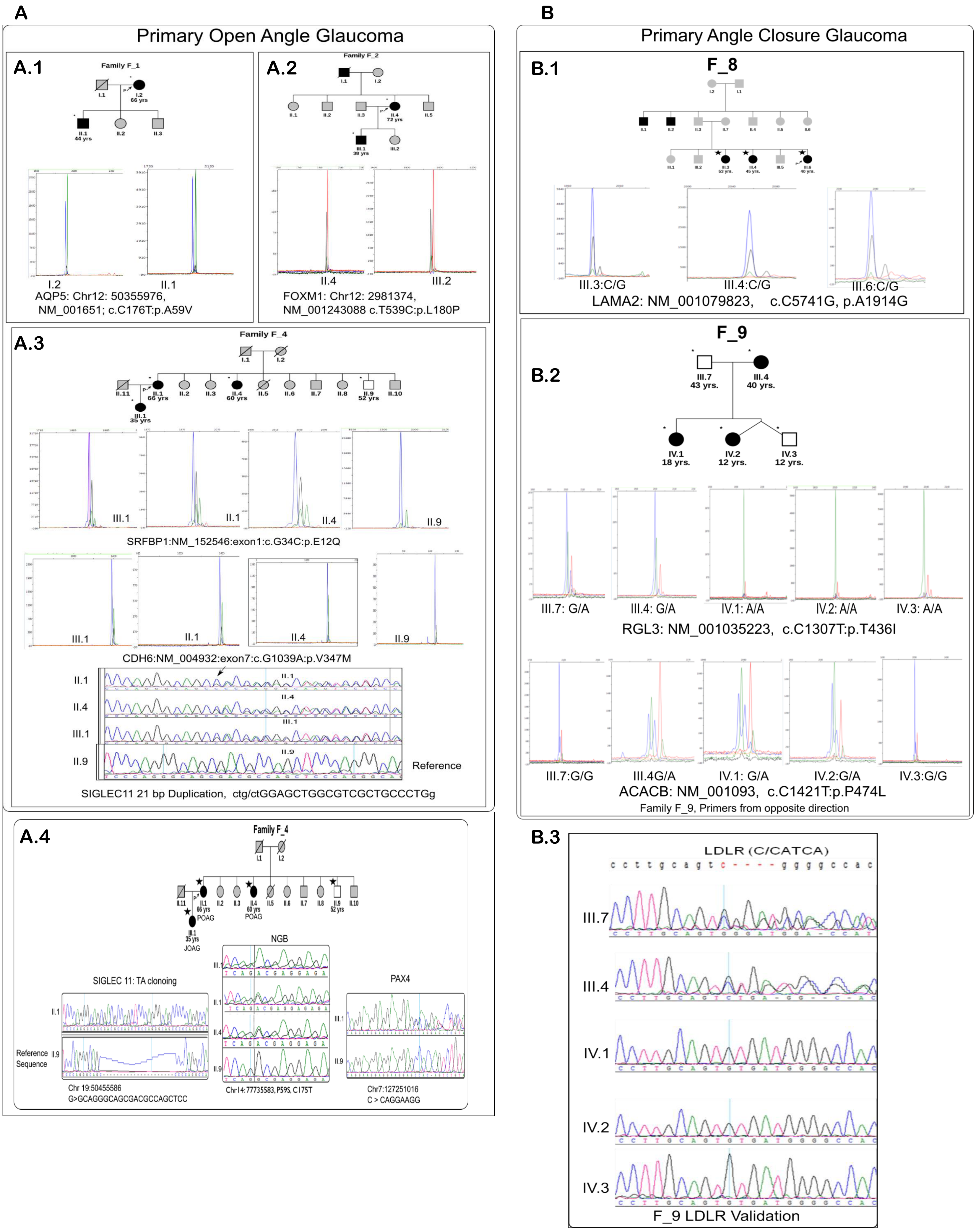

!"#$%&'(()%$*+,-.%
